# Supplementary material for: aPKC-mediated displacement and actomyosin-mediated retention polarize Miranda in Drosophila neuroblasts
Source: eLife. 2018 Jan 24;7:e29939. doi: 10.7554/eLife.29939 (PMC5783611; doi:10.7554/eLife.29939)
Supplement: Transparent reporting form [file elife-29939-transrepform.docx]

***eLife’s* transparent reporting form**

We encourage authors to provide detailed information *within their submission* to facilitate the interpretation and replication of experiments. If you have any questions, please contact us: [editorial@elifesciences.org](mailto:editorial@elifesciences.org).

**Sample-size estimation**

- You should state whether an appropriate sample size was computed when the study was being designed
- You should state the statistical method of sample size computation and any required assumptions
- If no explicit power analysis was used, you should describe how you decided what sample (replicate) size (number) to use

Please outline where this information can be found within the submission (e.g., page numbers or figure legends), or explain why this information doesn’t apply to your submission:

*No method has been use to compute appropriate sample size.*

*Sample sizes reported depended on the type of the experiment. Fixed samples analyzed by antibody staining allowed the collection of greater data point numbers. In cases where genetically mosaic tissue had been generated in which in addition the correct cell cycle stage had to be scored data point collection occurred at lower frequency.*

*A substantial fraction of experiments presented is based on live cell imaging. Neuroblasts are constantly dividing cells and their cell cycles cannot be synchronized. Understanding the dynamical changes occurring throughout the cell cycle requires therefore imaging over extended periods of time. In addition we imaged fluorescent reporters expressed from their endogenous promoters resulting in moderate to week expression of the reporter. Identifying cells that express sufficient levels to give enough contrast to sustain extended live imaging was therefor a further factor influencing sample size. We oriented us on figures in the literature in this regard used in the field.*

**Replicates**

- You should report how often each experiment was performed
- You should include a definition of biological versus technical replication
- The data obtained should be provided and sufficient information should be provided to indicate the number of independent biological and/or technical replicates
- If you encountered any outliers, you should describe how these were handled
- Criteria for exclusion/inclusion of data should be clearly stated
- High-throughput sequence data should be uploaded before submission, with a private link for reviewers provided (these are available from both GEO and ArrayExpress)

Please outline where this information can be found within the submission (e.g., page numbers or figure legends), or explain why this information doesn’t apply to your submission:

- *We have provided either in the main text, in the figures and/or in the figure legends how often an experiment was performed. The sample size n, that we provide reflects the total number of samples analyzed coming from 2 or more independent biological repeats. A definition of n in relation to technical and biological variation can be found on page 20, line 487.*
- *Sample sizes:*
- Figure 1A: sample size displayed in the figure.
- Figure 1B: Sample size found in quantification, Figure 1B’.
- Figure 2A: Sample size found in figure panel.
- Figure 2B: Sample size found in figure panel.
- Figure 2C: Sample size found in figure panel.
- Figure 2D: Sample size found in box plot, x axis.
- Figure 3A: Sample size detailed in quantification, found in 3C
- Figure 3B: Sample size detailed beneath the figure panels.
- Figure 4A: Sample size detailed in figure panels.
- Figure 4C/C’: Sample size detailed in graphs legend.
- Figure 4D/D’: Sample size detailed in graphs legend.
- Figure 5A: Sample size detailed in figure panels.
- Figure 5B: Sample size detailed in figure panels.
- Figure 5C: Sample size detailed in figure panels.
- Figure 5D: Sample size detailed in figure panels.
- Figure 5E: Sample size detailed on charts x axis.
- Figure 5F: Sample size detailed on chart.
- Figure 1 supplement 1: Sample size detailed beneath figure panels.
- Figure 1 supplement 2: Sample size detailed in figure legend.
- Figure 1 Supplement 3: Sample size detailed in figure panels.
- Figure 2 Supplement: Sample size detailed in figure panels.
- Figure 3 supplement: Sample size detailed in the figure. Quantification of fixed cells in panel A is found in the chart in A’.
- Figure 4 supplement: Sample size detailed in figure and figure legend.
- Figure 5 supplement: Sample size is detailed in the figure.

**Statistical reporting**

- Statistical analysis methods should be described and justified
- Raw data should be presented in figures whenever informative to do so (typically when N per group is less than 10)
- For each experiment, you should identify the statistical tests used, exact values of N, definitions of center, methods of multiple test correction, and dispersion and precision measures (e.g., mean, median, SD, SEM, confidence intervals; and, for the major substantive results, a measure of effect size (e.g., Pearson's r, Cohen's d)
- Report exact p-values wherever possible alongside the summary statistics and 95% confidence intervals. These should be reported for all key questions and not only when the p-value is less than 0.05.

*The statistical tests used are detailed in the figure legends where applicable and the p values are provided in the figures.*

Please outline where this information can be found within the submission (e.g., page numbers or figure legends), or explain why this information doesn’t apply to your submission:

(For large datasets, or papers with a very large number of statistical tests, you may upload a single table file with tests, Ns, etc., with reference to page numbers in the manuscript.)

**Additional data files (“source data”)**

- We encourage you to upload relevant additional data files, such as numerical data that are represented as a graph in a figure, or as a summary table
- Where provided, these should be in the most useful format, and they can be uploaded as “Source data” files linked to a main figure or table
- Include model definition files including the full list of parameters used
- Include code used for data analysis (e.g., R, MatLab)
- Avoid stating that data files are “available upon request”

Please indicate the figures or tables for which source data files have been provided:

We have submitted examples of representative live imaging of neuroblasts in form of downloadable movies.
